# A New Hope: Self-Assembling Peptides with Antimicrobial Activity

**DOI:** 10.3390/pharmaceutics11040166

**Published:** 2019-04-04

**Authors:** Lucia Lombardi, Annarita Falanga, Valentina Del Genio, Stefania Galdiero

**Affiliations:** 1 Department of Pharmacy, University of Naples Federico II, Via Mezzocannone 16, 80134 Naples, Italy; lucia.lombardi@unina.it (L.L.); valentinadelgenio@unina.it (V.D.G.); 2 Department of Agricultural Science, University of Naples Federico II, Via Mezzocannone 16, 80134 Naples, Italy; annarita.falanga@unina.it

**Keywords:** self-assembling, peptide, antimicrobial activity, nanomaterial

## Abstract

Peptide drugs hold great promise for the treatment of infectious diseases thanks to their novel mechanisms of action, low toxicity, high specificity, and ease of synthesis and modification. Naturally developing self-assembly in nature has inspired remarkable interest in self-assembly of peptides to functional nanomaterials. As a matter of fact, their structural, mechanical, and functional advantages, plus their high bio-compatibility and bio-degradability make them excellent candidates for facilitating biomedical applications. This review focuses on the self-assembly of peptides for the fabrication of antibacterial nanomaterials holding great interest for substituting antibiotics, with emphasis on strategies to achieve nano-architectures of self-assembly. The antibacterial activities achieved by these nanomaterials are also described.

## 1. Introduction

### 1.1. Antimicrobial Resistance and the Need for Novel Molecules to Substitute Antibiotics

History broadly documents cases of infectious diseases and witnesses that many pathogens were largely spread already in ancient Egypt and Greece (such as tuberculosis and diphtheria). At that time, people embraced a superstitious idea of diseases, and only much later did medical science reveal that many illnesses were caused by pathogens. At the beginning of the twentieth century, modern medicine brought an understanding of these pathologies and the discovery of antibiotics revolutionized the treatment of pathogenic diseases, saving millions of lives.

Many antibiotics available on the market are natural products of secondary metabolism of microorganisms and multicellular living beings; others are synthetic compounds usually deriving from natural molecules [1].

The pharmaceutical industry facilitated the immediate availability of antibiotics, which remain one of the most commonly prescribed classes of drugs. However, mass use (both for humans and animals) and easy access have also led to their overuse, prompting bacteria to develop resistance, which represents a serious problem for our society from both an economical and health perspective. As a matter of fact, diseases that were thought to be controlled by antibiotics are now resistant to these therapies. The antibiotics are ancient and ubiquitarious drugs in nature; thus, defense mechanisms against these antibiotics are as ancient as their production in nature; however, it is important to keep in mind that the number of resistant organisms is unparalleled [2], and clinically important bacteria are characterized by multiple drug resistance mechanisms (MDR) [3]. 

Resistance mechanisms involve the inactivation or modification of the antibiotics by enzymes, the protection or alteration of the antibiotic targets, the expulsion of the drug from the bacterial cells through efflux pumps, and the alteration of the cell membrane permeability by decreasing porin expression or expressing porin variants [4]. 

Another important issue is represented by biofilms. Pathogens are rarely in a free (planktonic) state, rather they form micro-colonies and produce biofilms to persist in a hostile environment [4]. Biofilm formation proceeds as a four-step process: (1) bacterial cells start attaching; (2) cells aggregate and accumulate forming multiple layers; (3) the biofilm matures and (4) cells detach from the biofilm into a planktonic state to produce a new biofilm elsewhere. Bacterial adhesion is the first and most important step. Bacteria adhere to each other and the surface on which the biofilm develops, exploiting van der Waals forces, hydrophobic interactions, as well as pili and fimbria, then they produce a layer of slime made of extracellular polymeric substances (EPSs), such as exopolysaccharides, proteins, extracellular DNA, and teichoic and lipoteichoic acids [5,6]. The slime protects the bacteria from antibiotic therapy, physiologic shear, and the host defense system. Host immune responses are often unable to eliminate bacteria growing in a biofilm because of the anaerobic environment created by bacteria and the slime, which makes immune cells less accessible. Subsequently, a chronic inflammatory response may be produced. Biofilms are also highly resistant to treatment with conventional antimicrobial therapies, which are not able to penetrate across the extracellular polymeric layer [7].

Great efforts are thus devoted to discovering novel molecules with different mechanisms of action to substitute antibiotics and prevent the return to a pre-antibiotic era. Antimicrobial peptides (AMPs) constitute a promising class of novel drug candidates, which may also be able to overcome pathogen resistance and, thus, represent excellent candidates for clinical exploitation [8,9]. Moreover, in this scenario, the development of self-assembled antimicrobial nanomaterials opens new avenues for addressing major resistance problems. Needless to say, the most recent literature reports on novel strategies and exploitation of nanoscience based technologies to produce on demand stimuli responsive antimicrobial compounds [10]. 

### 1.2. Biomedical Implants and Biofilms

Biomedical implants (prosthetics, catheters, and several other devices) have revolutionized medicine, but severe infections are associated with them because their surfaces are in contact with biological fluids and, thus, susceptible to bacterial colonization. For successful implantation, the rapid integration of biomaterials into host tissues is a key factor enabling the prevention of bacterial adhesion and colonization. It is impossible to create perfectly sterile wounds; thus, minor contamination of implant surfaces may be regarded as a physiological phenomenon. The implant surfaces become a reservoir of bacteria that can spread into the rest of the body, causing chronic infection. Additionally, several troubles are related to the development of biofilms on medical implants. The implant removal often represents the only chance to eradicate the biofilm and the increased number of replacement surgeries determines an increase in healthcare costs. If bacterial adhesion takes place before tissue regeneration occurs, the immune system often cannot prevent surface colonization and the subsequent formation of biofilm. Therefore, an important issue is prevention with inhibition of bacterial adhesion and development of sophisticated antibacterial implant materials. Infection-resistant materials can be obtained by: (a) modification of the biomaterial surface to confer anti-adhesive properties, (b) coating of the material with antimicrobial drugs, (c) combined coating with anti-adhesive and antimicrobial substances, (d) design of materials able to oppose biofilm formation after the initial bacterial attachment. Self-assembled monolayers (SAMs), use of various polymer-based materials [11], and liquid-infused nanostructured surfaces [12] are only a few examples of the various chemical approaches for coating an implant and generally, more than one mechanism of defense is required for a robust antimicrobial coating. As a matter of fact, a wide number of molecules that inhibit or destroy biofilms have been identified and used, often in combination, to prepare coatings for medical devices [13,14]. 

### 1.3. Antimicrobial Peptides

AMPs, alone or in combination with conventional antibiotics, are a particularly promising class of molecules with immunomodulatory activity [15], which constitute the first line of defense of all species against microbial invasion [8,16,17,18]. AMPs primary structure is generally characterized by a net positive charge (with some exceptions such as neutral or negatively charged peptides), able to elicit the initial electrostatic interactions with negatively charged microbial membranes or cell walls [16]. The presence of approximatively 50% hydrophobic residues renders the peptides amphiphilic and capable of folding on membrane contact to form α-helical or β-sheet-based secondary structures, thereby facilitating oligomerization (bacterial membrane disruption) and/or translocation through the microbial membrane (targeting of intracellular components) [16]. The mechanism of membrane disruption is often the predominant bactericidal mechanism but its exact role is still under investigation [19]. 

Effective antimicrobial peptides are characterized by high broad-spectrum activity; nonetheless, resistance mechanisms can be elicited and involve the alteration of membrane composition, peptidase expression, or peptide-efflux pumps. The risk to develop AMP resistance does not occur in the short term; as furthermore supported by their persistence in nature over millions of years [20]. Additionally, very little is known about AMP resistance mechanisms developed by biofilms. Biofilm resistance to AMPs appears to be mainly mediated by the interaction of the drugs with extracellular polymers, which are negatively charged; indicating that biofilm extracellular polymers may work by sequestration of AMPs [21].

Clinical and commercial development holds some other drawbacks, such as toxicity in some cases, susceptibility to proteases, and the cost of production; thus, extensive efforts are devoted to overcoming those drawbacks. Naturally-occurring AMPs provide templates for the design of molecules easier to produce and/or more potent; unusual amino acids or peptidomimetics are developed to avoid proteolytic degradation, while the obtainment of shorter peptides retaining activities represents a solution for the cost issue [18,22,23,24,25].

AMPs are commonly classified according to their structure: α-helical, β-sheet peptides, and extended/random-coil peptides [17]. The α-helical AMPs, including magainin, cecropin, pexiganan, temporins, and melittin are usually unstructured in aqueous solution but are able to adopt an amphipathic α-helical structure when interacting with biological membranes or membrane-mimicking environments. They are essentially cationic and amphipathic and are active against Gram-positive and Gram-negative bacteria and fungi [26,27,28,29]. Their activity is mainly attributed to the disruption of bacterial membranes. The β-sheet AMPs, such as α/β-defensins, and protegrin’s are stabilized by disulfide bridges, and form relatively rigid amphipathic structures, exerting their activities by disrupting bacterial membranes [30,31,32]. The third class comprises extended peptides with a broad spectrum activity, which are often rich in specific amino acid residues such as proline (such as proline-rich peptides originally isolated from insects), tryptophan and arginine (such indolicidin and tritrpticin), histidine (such as human salivary histatin) and lack secondary structure. They usually fold into amphipathic structures when in contact with a membrane and their activity is correlated both to membrane leakage and to interactions with intracellular targets through inhibition of nucleic acid synthesis, protein production, or other enzyme activities as cell-wall synthesis.

AMPs are often broad-spectrum antimicrobials and carry the risk of being toxic for eukaryotic cell and of annihilating the microbiota providing a suitable place for opportunistic pathogens. Because of their eventual toxicity for eukaryotic cells, many AMPs in clinical trials have been developed for topical use rather than systemic applications [33]. Recently, AMPs have been coupled to surfaces in order to overcome some complications of medical implants such as the formation of the biofilm [34]. When tethered on a surface, low amounts of AMPs are required for applications, and the peptides are spatially regulated with high local concentrations but limited systemic toxicity, and they might also be inherently more resistant to the protease attack [35,36,37]. 

Thus, AMPs represent an emerging strategy for dealing with bacterial infections. In depth understanding of the structure function relationship of AMPs will allow design and modifications of natural AMPs providing a new source of antimicrobial molecules. The use of supramolecular objects possessing antibacterial activity, which may be obtained by the self and co-assembly of AMPs appears extremely fascinating. This strategy may help in overcoming the drawbacks of traditional AMPs and enable the development of antimicrobial nanomaterials with improved stabilities and activities and sustained release. 

## 2. Self-Assembling in Nature 

Self-assembly refers to the spontaneous organization of molecules in ordered supramolecular structures thanks to their mutual non-covalent interactions without external control. The chemical and conformational structures of individual molecules carry the instructions of how these are assembled [38,39,40]. The same or different molecules may constitute the building blocks of a molecular self-assembling system [41,42], Generally, interactions are established in a less ordered state, such as a solution, random coil, or disordered aggregate leading to an ordered final state, which can be a crystal or folded macromolecule. The association of small molecules into well-ordered structures is driven by thermodynamic principles, thus, based on energy minimization. The interactions involved in the molecular assembly process are electrostatic, hydrophobic, hydrogen bonding, van der Waals interactions, aromatic stacking, and metal coordination [39,43]. Although non-covalent and individually weak (2–250 kJ mol^−1^), these forces can generate highly stable assemblies and govern the shape and function of the final assembly.

Self-assembly is essential for life, ubiquitous in nature and may represent a well of inspiration for material design and, thus, the development of novel and valuable supramolecular assemblies [44]. As a matter of fact, many biological structures possess a highly precise organization derived from specific interactions on a molecular scale, which are critical for their function. Examples are represented by nucleic acids to form the DNA helix [45], lipids in cell membranes [46], viral capsids [47], proteins which fold to have a secondary and tertiary structure or interact in trimer as it occurs with glycoproteins during viral fusion [48,49], and peptides such as antimicrobial peptides, which form toroidal pore or barrel-stave in bacterial membranes [8,50]. 

Self-assembling molecules provide the challenging opportunity to control chemical functionality and morphology and thus biological activity. Among the most multipurpose molecules with self-assembling properties are the peptides; in fact, careful design of the sequence can help in controlling folding patterns, while chemical modifications of side or main chains can provide chemical and physical functionalities.

### Basic Features of Self-Assembling Peptides

**Figure 1:**
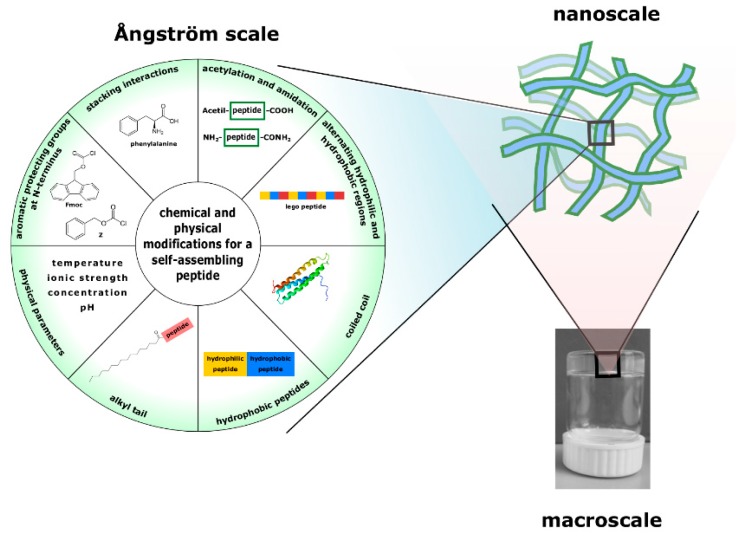
The appearance of a gel at various size scales. At the nanometric scale, the gel is a network of aggregates (usually fibers, tubes, etc.) that are formed thanks to spontaneous interactions among molecules (building blocks) of different nature. By changing chemically, the building blocks or the environmental parameters, the self-assembly can be controlled to obtain different on demand supramolecular nanostructures.

Peptides represent attractive self-assembling building blocks for construction of smart biomaterials with well-ordered structures and diverse functions capable of responding to environmental stimuli. Self-assembly of peptides is governed by noncovalent interactions, and varying the amino acid sequences and manipulating the environmental parameters, it is possible to modulate those interactions (Figure 1). Based on these forces, the self-assembly of peptides can be controlled to obtain diverse on demand supramolecular nanostructures (nanotubes, nanobelts, fibrils, nanovesicles, gels and nanocages), which can disassemble upon contact with the pathogen and release the cargo. 

Great attention has been devoted to the understanding of the driving forces regulating the self-assembly process because the different supramolecular nanostructures obtained are highly correlated to the different physical, chemical, and biological properties that can be achieved [51]. The obtained self-assembled nanostructures may possess functions improved by collective behavior, and show new properties and functions that are not owned by their building blocks or endowed with more functions by incorporation of new functional molecules.

Supramolecular materials for different applications can be obtained from pre-existing components termed building blocks, which can be atoms, small molecules, or macromolecules (bottom-up strategy) [52]. New building blocks can be designed from existing ones changing the chemical composition, the length, and the directionality of interactions to create new units, which contain all the necessary information that encodes their self-assembly. A self-assembled structure represents a situation of minimum energy and the environment creates a driving force that pushes the system to reach the thermodynamic minimum. By controlling environmental variables, the system reaches a new thermodynamic minimum leading to a different ordered structure [53]. In most cases, because the peptide self-assembly occurs by non-covalent interactions, their self-assembly is reversible and sensitive to the environment and the activity can be tuned controlling the association and the dissociation of the peptides.

Generally, the self-assembling ability of a peptide is mainly determined by the amphiphilicity of the molecule and the formation of secondary structures, which allow interactions among peptides. When dealing with cationic AMPs, due to their charges, self-assembling into a super structure is rarely observed in water solution; nonetheless, they are known to self-assemble and form fibrillar amyloid-like nanostructure or helical bundles in membrane environments; these structures allow them to exert their antimicrobial activity through an interaction with bacterial membranes [17]. When dealing with amphipathic AMPs in an aqueous solution, the polar residues prefer to be located on the surface of the aggregate while the apolar ones localize in the core; this arrangement could stabilize the peptide secondary structure further affecting the conformational transition taking place during the water-membrane interface interactions, which are usually important for the killing activity [54]. Moreover, AMPs could be modified chemically to introduce self-assembling properties in their sequence and influence the interaction between the AMPs and the cell membranes, which is key for their activity. This is a crucial factor to keep in mind when designing new self-assembled AMPs; in fact, the antimicrobial activity may be affected because the charge distribution and the secondary structure will be different from that of single peptide molecules. Nonetheless, in some cases, the antibacterial efficacy can be considerably enhanced when peptides are self-assembled into nanostructures [55], and their stability can be enhanced because they become less sensitive towards enzymatic degradation, renal filtration, and uptake by the reticuloendothelial system [56]. 

There are several strategies to confer self-assembling properties to peptides; one could be the chemical addition of a moiety, which provides the driving force necessary for the collapse into a nanostructure; modifications include the use of protected amino acids, small or long peptide sequences, lipids or alkyl tails. pH and ionic strength can be important factors for on demand control of peptide assembly and disassembly. Charged amino acids are key for pH-responsive self-assembled molecules; in fact, below a certain pH (at which they are neutral) they tend to self-assemble, while at a higher pH they will disassemble because of electrostatic repulsions. 

## 3. Amino Acids and Peptides as Building Blocks 

Some peptides adopt a specific secondary conformation and in the presence of appropriate stimuli or favorable physical conditions these secondary structures self-assemble to form nanostructures of different shapes, fibers, tapes, sheets, wires, ribbons, sphere, and have been produced and utilized for a variety of applications [57,58,59,60,61]. 

Peptides forming self-assembled aggregates can be very short such as mono-, di- and tripeptides usually N-terminally protected, but also longer and are able to produce different structural motifs such as α-helix, β-sheet, β-hairpin.

Xu et al. were the first to demonstrate that fluorenylmethyloxycarbonyl (Fmoc)-protected single amino acids, such as Fmoc-Lysine (Fmoc-Lys) or Fmoc-Valine (Fmoc-Val) or their mixtures were able to form fibers and hydrogels predominantly through π-π bonds [62,63]. Other research groups also reported the aggregation of Fmoc-amino acids, such as Fmoc-Phenylalanine (Fmoc-Val)and its fluorinated analogues [64,65]. Banerjee demonstrated that supramolecular structures can be also formed by co-assembling of different Fmoc-amino acids such as Fmoc-Lys and Fmoc-Glutamic acid (Fmoc-Glu), which display opposite charges [66].

Much interesting work was done on Fmoc-dipeptides. The first Fmoc-dipeptides (Fmoc-Leucine-Aspartic acid, Fmoc-Alanine-Aspartic acid, Fmoc-Isoleucine-Aspartic acid) for the development of supramolecular systems were studied and used by Janmey [67,68,69]. Gazit and co-workers reported noteworthy experiments performed on diphenylalanines assembling of tubular structures [70]. Gazit also described the assembly of Fmoc-Phe-Phe-COOH analogues, such as tert-butyloxycarbonyl (Boc)-Phe-Phe-COOH and benzoyloxycarbonyl (Z)-Phe-Phe-COOH in amyloid-like structures. In particular, Fmoc-Phe-Phe-COOH forms a material with the macroscopic characteristics of a gel at a high concentration in water solution and showing a higher rigidity than gel formed by longer peptides and stability across a broad range of physical conditions [71].

Hamachi et al. replaced Fmoc with a stimuli-triggered degradation unit, such as aryl-methoxycarbonyl (Armoc), p-nitrophenylmethoxycarbonyl (NPmoc), or 6-bromo-7-hydroxycoumarin-4-ylmethoxycarbonyl (Bhcmoc) [72]. The idea was that the removal of the hydrophobic group, which plays an essential role in the formation of self-assembled nanofibers in response to a specific stimulus, destroys the subtle balance of molecular interactions in the self-assembled nanofiber network, causing the gel-sol phase transition and the release of an incapsulated drug [72]. 

Banerjee et al. reported that Boc protected dipeptides, such as N-Boc-Leu-Phe, N-Boc-Phe-Leu, N-Boc-Leu-Leu, are able to form nanofibrillar networks in which the peptides assumed a β−sheet structure [64]. Those hydrogels were demonstrated to adsorb efficiently toxic dyes in water usually found in the industrial waste. 

However, self-assembling moieties may be also obtained attaching to peptides different aromatic components such as naphthalene. Adams et al. characterized a library of dipeptides conjugated to naphthalene, where they varied both the amino acids and the substituents present on naphthalene rings. They investigated the physicochemical properties (critical aggregation concentration, air-water partition coefficient at high pH and the apparent pK_a_) and gelation ability for each compound of the library to find a relationship between these properties and the predicted hydrophobicity of the overall conjugates [73]. 

Differently, from dipeptides, there are only a few examples of tripeptide based nanosystems [74]. Banerjee et al. reported three self-assembling tripeptide based systems with the common structure of Boc-Phe-X-Phe-OH, where X = Val, Leu, Phe.

Apart from the functionalization at the N-terminus with the Fmoc or other aromatic groups, another useful strategy to confer self-assembling properties to single amino acids, di- or tri-peptides is represented by the introduction of non-natural d-amino acid at the N-terminus. Examples are ^D^Val-Phe-Phe and ^D^Phe-Phe-Val reported by Marchesan [75]. 

Longer peptides were used to exploit their ability to produce α-helix, β-sheet, β-hairpin structural motifs to favor assemblies. The α-helix is a secondary structure stabilized by internal backbone hydrogen bonding; packing of α-helices through hydrophobic and van der Waals’ forces favors the formation of coiled-coil motifs and provides additional stabilization [76]. In the literature, there are few examples of coiled coil used to rationally design hydrogels [77]. Woolfson reported on several fibrous biomaterials based on α-helical dimers [78]. Such dimers were the building blocks to form helical fibrils in a controlled manner and a physical gel with a high content of water and the gel resulted to be temperature sensitive [78,79]. Indeed, gels with hydrogen-bonded networks (glutamine based) display sol-gel transition with an increase of temperature, whereas those with hydrophobic interactions (alanine based) strengthen when warmed. 

β-sheets consist of strands laterally connected by backbone hydrogen bonds, forming a commonly twisted sheet. β-sheets are associated with high-level aggregates, such as fibrils found in many human diseases, particularly Alzheimer’s disease. Many examples of β-sheet structures are reported in the literature. Boden and co-workers synthesized β-sheet tape as well as hydrogels [60,80]. A series of interesting β-sheet forming peptides is reported by Zhang [81]. These peptides, called lego peptides, are approximately 5 nm in size, have 16 amino acids and are characterized by a regular alternation of hydrophilic and hydrophobic amino acids and the charged residues used have an alternating positive or negative charge. They form stable β-sheet structures and nanofibers in water and hydrogel with the side chains partitioned into two sides, one polar and the other apolar. The most studied is Arg-Ala-Asp-Ala- Arg-Ala-Asp-Ala- Arg-Ala-Asp-Ala- Arg-Ala-Asp-Ala (RADA) and its self-assembly is driven by hydrophobic forces [82]. These peptides assume a random coil conformation in water but the increase of the ionic strength triggers self-assembly into β-sheets then self-supporting hydrogels [83]. 

The β-hairpin is also a secondary structural form, which is able to self-assemble, resulting in a hydrogel. These peptides, studied by Schneider and Pochan, are usually fully soluble and attain random coil conformation in solution; when an external stimulus occurs the peptide undergoes folding to β-hairpin and self-assembly forming a hydrogel [84].

## 4. Peptide Amphiphiles

Another key parameter that can be tuned to develop new self-assembled materials, is the amphipathic nature of the molecules. Peptide sequences with high assembling ability could be designed containing both hydrophilic and hydrophobic residues (peptide amphiphiles) or through the addition of an amphiphilic peptide bound at C or N-terminus of the native sequence. 

An example of amphiphiles is polyelectrolytes such as polylysine or polyglutamate connected to hydrophobic domains, such as polyleucine or polyvaline [85]. The hydrophilic–lipophilic balance is a controlling factor for the assembly and co-assembly process of peptide amphiphiles, as a decrease in the hydrophobic content does not allow the peptide to attain the desired nanostructure [86]. These peptides are also named lipid-like peptides because they have an either positively or negatively charged head group, and a hydrophobic tail consisting of six hydrophobic amino acids. They are approximately 2 nm and can self-assemble into nanotubes and nanovesicles and examples are reported by Zhang group [81]. 

Another category of peptide amphiphiles is represented by alkylated or lipidated peptides [87]. Connecting a hydrophobic tail to a hydrophilic peptide adds hydrophobicity to the peptide, facilitating its self-organizing ability. Such peptides self-assemble into micelles or bilayer structures in a concentration dependent fashion and increased concentrations lead to nanofibers or tube formation, which can further self-assemble to form a 3D hydrogel [88]. 

It is also possible to use more than one motif at the same time as done by Stupp and co-workers [87]. They reported peptide amphiphile bearing five different structural and functional domains: a long alkyl chain (hydrophobic character), a cysteine-rich region (disulfide bond formation), a linker made of glycine residues (flexibility), phosphorylated serine (interaction with calcium ions and mineralization) and an Arg-Gly-Asp motif (cell targeting). Self-assembly of this peptide is triggered by different forces, such as stabilization by van der Waals and hydrophobic forces, covalent bonds, ionic bridging, hydrogen bonding, and counter-ion screening. These features together with concentration significantly influence self-assembly and gel mechanical properties [89]. 

It is right to point out that not only chemical modifications can attribute self-assembling properties to the peptides but also changes in the surrounding environment (solvent, temperature, ionic strength, pH) can lead to supra-molecular structures from peptides that normally do not aggregate. All the motifs described above can be used to confer self-assembling properties to antimicrobial peptides.

## 5. Reports on Self-Assembling Systems with Antimicrobial Properties 

In this paragraph, we will describe several examples of self-assembled AMPs, which will include, self-assembled peptide nanosystems with appeared antimicrobial activity, AMPs with the ability to self-assemble usually in the membrane and naturally-occurring AMPs modified with a self-assembling moiety.

The self-assembling properties of a peptide are essentially correlated to their secondary structure and amphiphilicity, which means that de novo design may assure the self-assembling ability but not the antimicrobial activity; on the contrary, sequences with antimicrobial activity are usually also characterized by amphiphilicity and by their ability to assume α-helical or β-sheet structures, which are the key features for self-assembling and may be exploited for the design of self-assembling antimicrobial materials, which unfortunately may lose their activity in the self-assembling process, which is true both for AMPs with the ability to self-assemble usually in the membrane and naturally-occurring AMPs modified with a self-assembling moiety. Moreover, only a few studies are reported that address from a biophysical point of view the structure-function relationship of surface immobilized AMPs. A further issue is that each system is different and the technology used for the immobilization (in the cases addressed by this review, the self-assembling moiety used) may lead to varied structures and thus activities. A nice paper by Xiao et al. compares surface immobilized AMPs (cecropin and melittin) on different surfaces by spectroscopic methodologies and finds that immobilized AMPs may kill bacteria with a different mode of action compared to free peptides in solution [90].

Therefore, even though sequences with known antimicrobial activities are preferred, their activity is not necessarily retained, and the self-assembling process is also complicated, and most predictions could fail in actual experiments. The tools described in the previous paragraphs have all been used to assist in the self-assembling of AMPs [10,91]. 

Of great clinical interest are the ultrashort aromatic dipeptides, which often consist of two phenylalanines conjugated to a molecule of high aromaticity, such as naphthalene (Nap) or Fmoc. NapPhe-Phe [92] and FmocPhe-Phe [93]; possess several key properties making them an ideal self-assembling biomaterial platform and were also shown to have antimicrobial activity. The diphenylalanine nano-assemblies were proved to inhibit bacterial growth and damage bacterial morphology at a concentration of approximatively 125 μg/mL [94]. This is a minimal model of an antibacterial material and supports the development of more potent self-assembled nanosystems. 

Porter et al. reported on the antibiofilm activity of diphenylalanine peptide nanotubes. These self-assembled nanostructures present sufficient antibacterial activity to eradicate mature biofilm forms of bacteria widely implicated in hospital infections [95].

For instance, Marchesan et al. synthetized a tripeptide (^D^Leu-Phe-Phe), which yields physical hydrogel at physiological pH [96]. That hydrogel was demonstrated to be able to carry a drug (ciprofloxacin) and release it in a controlled fashion. This approach of drug incorporation into the nanostructure of a simple tripeptide hydrogel by self-assembly may have important applications for novel antimicrobial coatings [96]. 

Many self-assembling antimicrobial peptides are not derived from natural sequences but are synthetic peptides able to self-assemble and display activity against pathogens. In general, the head may possess positively or negatively charged residues while the hydrophobic portion contains amino acids such as Val, Ile, Leu, Phe, Trp (tryptophan), or Tyr (tyrosine).

Peptide amphiphiles of the generic composition X_m_Z (m = 3, 6 or 9; X = hydrophobic residue; Z = positively charged residue) displayed antimicrobial activities like natural antimicrobial peptides; this is supported by evident permeation and disruption of the bacterial membranes. As the length of peptide hydrophobic tail usually made of a sequence of alanines increased, the extent of membrane penetration and the ability also amplifies; confirming the correlation between the propensity for self-assembly and their membrane penetration power/antimicrobial capability [97]. 

Xu et al. recently reported the design of antimicrobial nanofibers based on the general formula Trp-(Lys)_x_-(Gln-Lys)_y_-(Lys)_z_. Protease stability, cytotoxicity, and antimicrobial activity could be tuned by adjusting the ratio between the different blocks. The obtained compounds reflect an energetic balance between the intermolecular hydrogen bonding and hydrophobic interactions among the (Gln-Lys) repeating units and electrostatic repulsion among the lysine residues [98,99]. 

Goel et al. reported on a short self-assembling amphiphilic mixed α/β pentapeptide; in comparison to peptides containing only α amino acids, mixed peptide present greater chemical and physical diversity, excellent stability and activity. In particular, this pentapeptide (H-Lys-βAla-βAla-Lys-βAla-OEt) was active against both Gram-positive and Gram-negative bacterial strains [100].

Another strategy is the conjugation of alkyl lipid chains of different length and/or hydrophobic amino acid residues. The attachment of these moieties usually leads to β-sheet conformation and the self-aggregation into fibrils [87]. 

Heparin-binding Cardin-motif amino acid sequence ((Ala-Lys-Lys-Ala-Arg-Lys)_2_) is a designed peptide, which self-assembles into cylindrical supramolecular structures thanks to hydrophobic interactions of the hydrophobic palmitic tail groups. The aggregation is directed into cylindrical shapes by (Val)_4_-(Lys)_4_ peptide forming the β-sheet structure [101]. 

Mitra et al. studied a series of lipopeptides C_16_−Lys-X-Lys (X is Ala, Gly, Leu, or Lys), potent against both bacteria and fungi, with C_16_−Lys-Lys-Lys being the best antimicrobial compound [102]. 

In nature, there are examples of lipopeptides, such as surfactins, iturins, and lichenysin, which have antifungal activity [103]. Surfactin is produced by *B. subtilis* bacterium and is able to aggregate into spherical micelles in bulk aqueous solution as shown by biophysical experiments (SANS, SAXS and, Transmission electron cryomicroscopy) while the aggregates assume a globular shape when at the air/water interface [104]. Another lipopeptide, mycosubtilin, forms aggregates of a different shape, nanotapes [104]. As said, most used strategies to promote the aggregation of AMPs were inspired by nature. Many scientific articles report that the addition of a lipid moiety in the antimicrobial peptide structure confers self-assembling properties and may also enhance anti-infective properties. An example is represented by TAT, a peptide (Tyr-Gly-Arg-Lys-Lys-Arg-Arg-Gln-Arg-Arg-Arg) derived from the HIV glycoprotein. TAT is used as antimicrobial peptide against drug-resistant bacteria, yeast, and fungi, and transporter of other peptides, proteins, nanoparticles, or anticancer drugs [105,106]. Studies demonstrate that the conjugation of TAT to poly-arginine sequence, three glycine residues as spacer and cholesterol (Chol-(Gly)_3_-(Arg)_6_-TAT) improves the transport and antimicrobial strength, as well as allows this peptide to assemble into micelles (CAC of 10 μM in deionized water), which are very effective against *S. aureus* in vivo [107]. 

An interesting approach was recently used by Huang et al. [108]; they exploited the self-assembling properties of the peptide RADA16 [109] to prepare nanofibers of RADA16-AMP with antibacterial activity. The antibacterial peptide used is Tet213 (Lys-Arg-Trp-Trp-Lys-Trp-Trp-Arg-Arg-Cys) and they found that it was retaining its activity against *S. aureus* when self-assembled with RADA16.

Liu et al. reported about stimuli-responsive self-assembled peptides made from the antibacterial peptide (Lys-Ile-Gly-Ala-Lys-Ile)_3_-NH_2_ [110]. This short de novo designed peptide consists of a central tetrapeptide linker flanked by two antibacterial peptide sequences that convert to β-sheets when exposed to external stimuli. The balance between electrostatic repulsion and hydrophobic attraction determines the molecular state and assembly and disassembly of the designed peptide and is responsible for the phase transition of the molecules and formation and growth of individually dispersed nanofibers. When exposed to stimuli such as pH, ionic strength, and heat, the peptide is capable of undergoing a reversible transition from a random coil to a β-folded structure and further self-assembly into a hydrogel whose surface is essentially holding the antibacterial activity [110]. The active AMP sequence was here used as a module, and the strategy was to combine the properties of an AMP with those of a self-assembling sequence. The central tetrapeptide linker allows for the formation of the β-sheet structure and the AMP sequences were located on the external surface of the fiber.

The presence of the AMP sequence on the periphery of the nanofiber increases their effective local concentration compared to soluble peptides and is the driving force for improved antibacterial activity. A novel versatile platform was developed in our laboratory to immobilize one AMP (but the same strategy can be exploited to immobilize several AMPs) on a peptide based biomaterial [111]. As proof of concept, WMR (H-Trp-Gly-Ile-Arg-Arg-Ile-Leu-Lys-Tyr-Gly-Lys-Arg-Ser-NH_2_), previously identified as a modification of the native sequence of the marine antimicrobial peptide myxinidin, was used [18,22,23,24]. The fiber structure was obtained through a self-assembling peptide module and a hydrophobic chain, while the external surface of the fiber was decorated with WMR [111]. The self-assembled nanostructures also provide a mean to increase stability and half-life. The multivalent presentation of WMR on self-assembled nanostructures improved anti-biofilm activity against the Gram-negative bacterium *Pseudomonas aeruginosa* and the fungus *Candida albicans*. Interestingly, fibers were able both to inhibit the biofilm formation and to eradicate pre-formed biofilms with both processes being key for biomedical applications [111]. This seems to be a sound strategy to design smart materials, which may also contain a conventional antibiotic and be stimuli responsive (pH-driven), releasing the loaded antibiotic and AMPs following a change in pH. 

Hong et al. recently reported the use of bacitracin A modified with poly(d,l-lactic-*co*-glycolic acid) (PLGA) and polyethylene glycol (PEG) as promising antibacterial compounds able through self-assembling to increase the local concentration of the active molecule and resulting in a stronger antibacterial potency. They clearly proved the potential of designed self-assembled peptide molecules deriving from naturally-occurring antibacterial scaffolds for future therapeutic applications [56].

**Figure 2:**
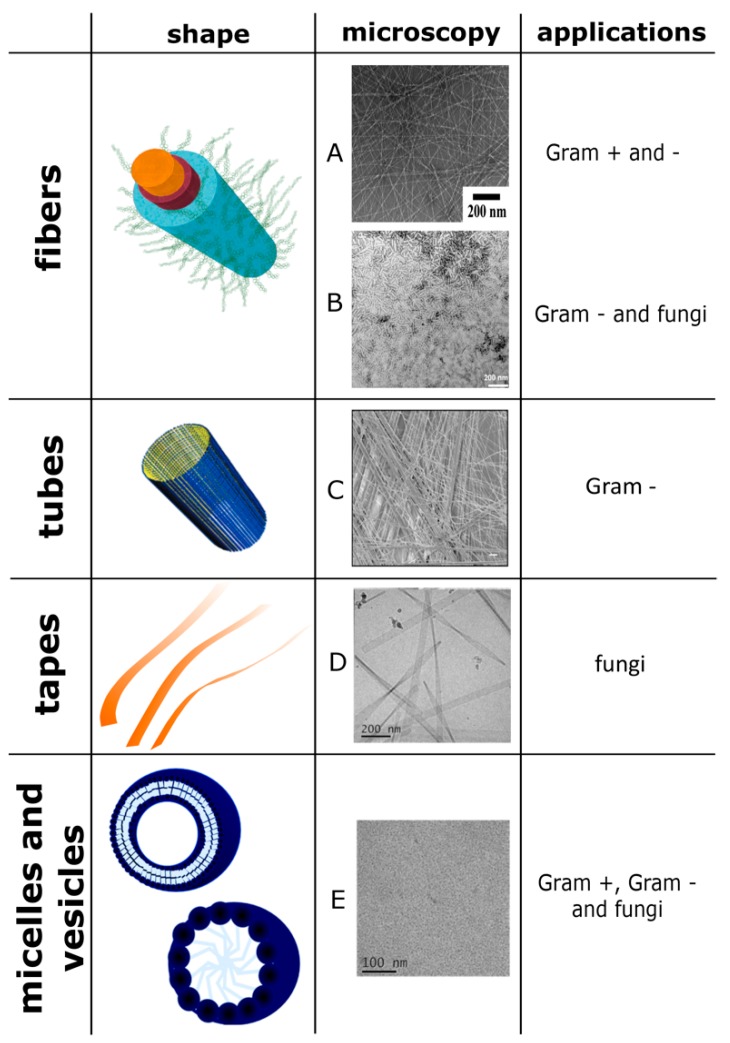
Some of the structures with antimicrobial properties mentioned in the article and their respective shapes assumed after the self-aggregation and their applications on pathogens. The image A is reprinted (adapted) with permission from reference [92]. Copyright 2014 American Chemical Society. The image B and the image of the fiber are reprinted (adapted) with permission from reference [111], scale bar is 200 nm. Copyright 2019 American Chemical Society. The image C is reprinted (adapted) from reference [94], scale bar is 10 μm. This article is licensed under a Creative Commons Attribution 4.0 International License http://creativecommons.org/licenses/by/4.0/. The images D and E are reproduced from reference [104]—Published by The Royal Society of Chemistry. The images of the tube are reprinted (adapted) with permission from reference [112] Copyright 2009 American Chemical Society. The images of micelle and vesicle are reproduced from reference [42] published in 2017 by The Royal Society of Chemistry.

Figure 2 reports some of the structures with antimicrobial properties mentioned above and their respective shapes assumed after the self-aggregation.

## 6. Conclusions and Perspectives

Self-assembly is key to life being widely exploited in nature especially for the functionality of living cells [113]. Thus, self-assembly represents a fascinating strategy to take advantage of non-covalent interactions and combine different elements on a single nanosystem, which may find applications for a number of nanotechnological purposes. It is also recently emerging in the biomedical field thanks to its good biocompatibility, design flexibility, and easy modification by functional groups [41,114]. 

Nowadays, the incorporation of antimicrobial peptides into artificial materials has become an effective strategy to improve the surface properties of materials for many applications. The food industry is highly interested in the development of self-assembled AMPs that avoid the development of resistance in bacteria and fungi without acting on specific targets. The development of self-assembled systems for the delivery across the blood brain barrier of AMPs and antibiotics is also a key approach to treat brain infections. Self-assembled peptide nanosystems may also have applications in vaccine design. Another interesting usage is the strategy of combining more than one active molecule to improve the antimicrobial spectrum of activity and potency but also to endow the nanosystem with extra activities providing the opportunity to design smart materials. Self-assembled/co-assembled systems with AMPs and chemotherapeutic drugs may represent an appealing strategy also for cancer [115].

In this framework, integrating antimicrobial peptides in or on the surface of a self-assembling systems offers the opportunity to engineer their antimicrobial activity against a wide range of Gram-positive and Gram-negative bacteria while reducing hemolysis and allergic responses and potentially the development of resistance, establishing an innovative design principle for the development of antibacterial materials. Moreover, particularly attractive, is the possibility to combine multiple components, including AMPs and conventional antibiotics, which may open new avenues in reducing both the administration dose of the antibiotic and the development of resistance. The integration of AMPs on self-assembling peptide nanostructures represents a novel strategy to improve biocompatibility by reducing toxic effects for human cells and enhancing toxicity for bacterial cells. The multivalent presentation of AMPs on the surface of self-assembled supramolecular nanostructures provides significant improvements compared to the activity of single soluble peptides because it can aid not only in increasing stability and half-life of peptides but also in augmenting and controlling the local concentration of the active peptide enabling enhanced interactions.

This systematic investigation will help to further validate the self-assembling platforms to re-engineer thousands of natural and synthetic AMPs, boosting their therapeutic potential. Self-assembled AMPs represent promising candidates in the field of pharmaceutical sciences and biomedical engineering, even though certain drawbacks still have to be solved to achieve commercial use. Nonetheless, they represent a huge fortune that cannot be neglected.
